# Survival rate of diabetic-related lower extremity amputees in hospitals in the Eastern Cape

**DOI:** 10.4102/ajod.v13i0.1503

**Published:** 2024-11-15

**Authors:** Aviwe S. Mgibantaka, Alfred Musekiwa, Moleen Zunza

**Affiliations:** 1 Division of Epidemiology and Biostatistics, Faculty of Medicine and Health Sciences, Stellenbosch University, Stellenbosch, South Africa; 2 Department of Rehabilitation Medicine, Faculty of Medicine and Health Sciences, Walter Sisulu University, Mthatha, South Africa; 3 School of Health Systems and Public Health, Faculty of Health Sciences, University of Pretoria, Pretoria, South Africa; 4 Department of Global Health, Faculty of Medicine and Health Sciences, Stellenbosch University, Stellenbosch, South Africa

**Keywords:** mortality, diabetes mellitus, diabetic foot, lower extremity amputations, comorbidities

## Abstract

**Background:**

Diabetes mellitus (DM) is a global health concern that has greatly affected South Africa. The gap in the current management of DM has resulted in complications such as lower extremity amputations (LEAs) and death. Eastern Cape province reflects this struggle, with disparities in access to healthcare and poor health outcomes. Understanding survival rates and associated factors between the urban Livingstone Hospital and the rural Nelson Mandela Academic Hospital can improve health interventions and outcomes.

**Objectives:**

This study compared the survival rate of patients in urban areas and those in rural areas.

**Method:**

This retrospective cohort study was conducted in an urban and a rural hospital by reviewing existing medical records of diabetic patients who underwent an LEA between 2016 and 2019.

**Results:**

The retrospective cohort study examined 439 diabetic-related LEA cases. This study found that residing in rural areas significantly decreased the risk of mortality by 62% compared with living in the urban areas. Factors such as haemoglobin A1c (HbA1c) levels, nephropathy, cardiovascular disease, human immunodeficiency virus (HIV), other comorbidities and level of amputation significantly influenced survival probabilities.

**Conclusion:**

Survival analysis indicated a significant difference in the 3-year survival probabilities of the two groups, favouring rural residency (*p* = 0.001). The biggest cause for concern between the two regions was uncontrolled blood glucose levels as this resulted in high mortality rates.

**Contribution:**

Insights from this study have shown that introducing podiatry and orthotics at primary healthcare (PHC) could improve foot care and reduce diabetic-related LEAs and mortality.

## Background

Diabetes mellitus (DM) is a chronic condition that requires interminable medical care to hinder the development of its complications. In recent years, there has been a rapid upsurge in the prevalence of DM in underdeveloped and developing countries. In South Africa, the prevalence of diabetes has almost tripled from 4.5% in 2010 to 12.7% in 2019 (Grundlingh et al. 2022).

Diabetes-related mortality has increased in South Africa, from 5.15% in 2014 to 5.5% in 2016 (Mtshali & Mahomed 2020). According to Lee et al. (2020), the prevalence of diabetes is increasing because of the ageing population, lack of exercise, unhealthy diets, population growth and increased body mass index (BMI), thus resulting in a rising incidence of diabetic foot (DF). Results from the South African National Health and Nutrition Examination Survey (SANHANES-1, 2011–2012), the country’s first national survey on non-communicable diseases revealed that just half (51%) of patients with diabetes on treatment had controlled blood glucose (HbA1c < 7%; Mtshali & Mahomed 2020). Approximately 2.5% of patients with diabetes develop DF annually, and 15% are predicted to be at risk of developing DF during their lifespan (Namgoong et al. 2016). Diabetic foot is defined as infection, ulceration or destruction of tissues of the foot of a person with, or previously diagnosed with, diabetes (Shabhay et al. 2021) and is the leading cause of infection, hospitalisation and diabetic-related lower extremity amputations (LEAs).

Furthermore, it is estimated that up to 80.6% of people living with diabetes (diagnosed and undiagnosed) in South Africa have an unfulfilled foot care need, especially at the primary healthcare (PHC) level (Ntuli & Letswalo 2023). Diabetic patients need aggressive foot care and screening to avoid amputation and prevent the progression of the disease (Khan et al. 2020; Shabhay et al. 2021). Patients with DF are not only vulnerable to LEAs but also a reduced life expectancy, with both hospital- and community-based studies reporting a 5-year survival rate of approximately 55% (Lin et al. 2021).

The survival rate in diabetic patients who have developed LEAs is poorer than that of cancer patients (Lin et al. 2021). The high number of patients with uncontrolled diabetes worsens the effects of the rapidly increasing diabetic burden on health systems (Mtshali & Mahomed 2020). Management of diabetes and associated complications accounted for 12% of global healthcare expenditure in 2015, and it was forecasted that this figure would increase to 19% of the global healthcare expenditure by 2040 (Mtshali & Mahomed 2020). In South Africa, 8.5% of the country’s gross domestic product (GDP) is allocated to healthcare, 3.5% more than the recommended healthcare expenditure per country by the World Health Organization (Ntuli & Letswalo 2023).

There are many factors associated with diabetic-related LEAs. These include gender, duration of diabetes, age at diabetes diagnosis, poor glycaemic control, diabetes-related microvascular complications (neuropathy, nephropathy, retinopathy), insulin therapy, body mass index, smoking, lipid abnormalities and prior amputation. Furthermore, diabetic-related LEAs are a major complication negatively affecting patient survival and quality of life (Wiessman et al. 2015) and have significant health and socioeconomic consequences with unfavourable effects on the quality of life (Namgoong et al. 2016). Lower extremity amputations increase the economic burden on the healthcare system of a country, as they result in loss of income and independence of individuals and increase morbidity and mortality.

South Africa uses a referral healthcare system, with PHC being first-level entry into the South African healthcare system. This is where patients receive standard care focussing on disease prevention, health promotion and referral to higher and more advanced levels of care, should the need arise (Ntuli & Letswalo 2023). People living with diabetes receive monthly chronic treatment and 6-month blood glucose monitoring at PHC (Ntuli & Letswalo 2023).

There are limited data on survival experiences of diabetic patients with LEAs. We conducted a retrospective study comparing the survival rates of patients with LEA, at Livingstone urban hospital and Nelson Mandela Academic rural hospital (NMAH) and explored factors associated with 3-year survival rates. Study findings may inform allocation of medical resources and targeted management and monitoring of diabetic patients with LEAs at higher risk of mortality.

## Research methods and design

### Study design

We conducted a retrospective cohort study reviewing medical records of adult patients who had undergone diabetic-related LEAs in Livingstone Hospital and NMAH between 01 January 2016 and 31 December 2019.

### Study setting

The study was conducted at NMAH in Mthatha and Livingstone Hospital in Port Elizabeth (Gqeberha). NMAH is located in the eastern region of the Eastern Cape province (former Transkei homeland) and provides services mainly to the rural parts of the province. Livingstone Hospital services the urban population of the western region of the province. The tertiary hospitals provide surgical, post-trauma counselling, physiotherapy and occupational services.

### Sample size estimation

The sample size calculation was done using Stata version 17.0 Statistical Software (College Station, TX: StataCorp LLC). A study by Beeson et al. (2023) reported diabetic-related lower extremity amputee mortality rate in an urban setting of 19.15% at 5 years, and a study by Brennan et al. (2022) recorded rural mortality rates to be at 28% at 5 years. We assumed similar mortality rates at 3 years to detect a mortality rate difference of 8.85% between the urban and rural hospitals. Using the Stata command, power two proportions 0.1915 0.28, test (chi-squared), a sample size of 360 in each group (total of *n* = 720) was required, to achieve power of 80% at the 5% significance level. To account for missing data, the sample size was inflated by 15%, 424 patients for each group.

### Study population

We included adult female and male patients (18 years and above) at Livingstone Hospital and NMAH, with diabetic-related LEAs and surgical debridement. Patients with amputation distal to and including disarticulation of the hip were included.

Patients with non-diabetic and traumatic-related amputations were excluded from the study. During the peak of the coronavirus disease 2019 (COVID-19) pandemic, a lot of elective surgeries were rescheduled as the focus had shifted to the pandemic; therefore, we excluded patients presenting at the hospitals in year 2020.

### Study procedures and measurements

We retrospectively reviewed medical records of patients at two tertiary hospitals in the Eastern Cape province, South Africa. We extracted data from 01 January 2016 to 31 December 2019 of patients meeting eligibility criteria, using discharge summaries and the Health Management System 2 (HMS2) database. We extracted data of eligible patients with complete treatment data in discharge summaries. A discharge summary is a detailed report on the biographical details of the patient, referring practitioner details, hospital details, clinical diagnosis on admission, investigations (blood tests), imaging, final diagnosis during the patient’s stay at the hospital, patient management, complications suffered by the patient, procedure performed on the patient, treatment (medication) on discharge, condition of patient on discharge, future management with explanations for changes and final disposition.

The Eastern Cape Department of Health uses the HMS2 for electronic storage of patient clinical data. Currently, the HMS2 database records patient biographic information, all admissions at hospitals at different times, outpatient department visits and the status of each patient as being alive, unknown and died. Unknowns are present when the final disposition has not been recorded on the discharge summary. Patient statuses are updated every midnight.

We extracted sociodemographic characteristics, including age in years, gender (male or female), marital status (single or married), employment status (employed or unemployed) and residence (rural or urban). Clinical characteristics included HbA1C percent (%) diabetic-related macrovascular and microvascular complications (neuropathy/nephropathy/retinopathy), insulin therapy, previous amputations and level of amputation, diagnosis on admission, length of hospital stays, transfer and/or referral of patients to other departments or specialities during admission, foot ulcer classification (Meggit–Wagner classification), surgical operation(s) and final disposition. The outcome variable was time to all-cause mortality, from the date of amputation.

### Statistical analysis

Stata version 18.0 was used for statistical analysis. Continuous variables were summarised using the mean (standard deviation [s.d.]) or median (range). Categorical variables were summarised as count (percent). The chi-squared test or Fisher’s exact test was used to test associations between categorical variables. The *t*-test was used to compare means between the two groups.

The Kaplan–Meier method was used to estimate survival probabilities. The Kaplan–Meier curve was used for visual display of survival probabilities. We used the log-rank test to compare the survival distributions of diabetic-related lower extremity amputees in the urban Livingstone Hospital and the rural NMAH. We explored factors associated with 3-year mortality using Cox regression model, and geographical location was the exposure of primary interest. We reported hazard ratios as measures of association with corresponding 95% confidence interval (CI). Variables with a *p* < 0.1 in the bivariate Cox regression analysis were included in multivariate Cox regression model. Significance level was set at *p* < 0.05 in the multivariate Cox regression.

### Ethical considerations

Stellenbosch University Health Research Ethics Committee (HREC) approved the study (Reference No: S23/06/144). Eastern Cape Department of Health granted permission to conduct the research at Livingstone Hospital and NMAH (EC 202309_002). Participants were assigned a unique identifier study number to protect the identity of the participants.

## Results

We included 396 patients from Livingstone Hospital and 43 patients from NMAH, much less than the sample size estimated for this study because of medical files with incomplete data and missing patient files.

### Sociodemographic characteristics

The mean age of the participants was similar in both the groups, 60.9 years in Livingstone Hospital, 59.2 years at NMAH and the overall mean age was 60.7 years. Male participants were dominant (69.9%). Many of the participants were married, divorced or widowed. More than 70% of participants were unemployed (Table 1).

**TABLE 1:** Sociodemographic characteristics of study participants (*N* = 439).

Characteristic	Livingstone Hospital: *N* = 396				NMAH: *N* = 43				Total: *N* = 439			
	Mean	s.d.	*n*	%	Mean	s.d.	*n*	%	Mean	s.d.	*n*	%
**Age in years**	60.9	10.4	-	-	59.2	12.5	-	-	60.7	10.7	-	-
**Gender**												
Male	-	-	269	68.8	-	-	38	79.2	-	-	307	69.9
Female	-	-	122	32.2	-	-	10	20.8	-	-	132	30.1
**Marital status**												
Single	-	-	32	8.2	-	-	8	16.7	-	-	40	9.1
Married (divorced and widowed)	-	-	359	91.8	-	-	40	83.3	-	-	399	90.9
**Occupation**												
Employed	-	-	98	25.3	-	-	10	20.8	-	-	108	24.8
Unemployed	-	-	291	74.7	-	-	38	79.2	-	-	329	75.2
**Geo-location**												
Urban	-	-	389	99.5	-	-	7	14.6	-	-	396	90.2
Rural	-	-	2	0.5	-	-	41	85.4	-	-	43	9.8

s.d., standard deviation; NMAH, Nelson Mandela Academic Hospital.

### Clinical characteristics

Participants clinical characteristics are presented in Table 2. The overall mean (s.d.) HbA1c was 11.4% (3.8%). The mean HbA1c in the Livingstone Hospital and NMAH samples were 11.4% (3.9%) and 11.6% (3.2%), respectively. More participants had neuropathy in the Livingstone Hospital population (21%) than those in NMAH (7%; *p* = 0.03).

**TABLE 2:** Summary statistics for clinical profiles of patients and health system factors.

Characteristic	Total						Livingstone Hospital: *n* = 396						NMAH: *n* = 43						*p*
	Mean	s.d.	Median	Range	*n*	%	Mean	s.d.	Median	Range	*n*	%	Mean	s.d.	Median	Range	*n*	%	
HbA1C %	11.4	3.9	-	-	-	-	11.4	3.9	-	-	-	-	11.6	3.2	-	-	-	-	0.71
Length of hospital stay (in days)	-	-	6	1–48	-	-	-	-	6	1–48	-	-	-	-	9	3–31	-	-	0.002 *
**Macrovascular:**	-	-	-	-	-	-	-	-	-	-	-	-	-	-	-	-	-	-	0.74
Yes	-	-	-	-	438	99.8	-	-	-	-	395	99.8	-	-	-	-	43	100	-
No	-	-	-	-	1	0.2	-	-	-	-	1	0.3	-	-	-	-	0	0	-
**Neuropathy:**	-	-	-	-	-	-	-	-	-	-	-	-	-	-	-	-	-	-	0.03*
Yes	-	-	-	-	86	19.6	-	-	-	-	83	21.0	-	-	-	-	3	7	-
No	-	-	-	-	353	80.4	-	-	-	-	313	79.0	-	-	-	-	40	93.0	-
**Nephropathy:**	-	-	-	-	-	-	-	-	-	-	-	-	-	-	-	-	-	-	0.21
Yes	-	-	-	-	14	3.2	-	-	-	-	14	3.5	-	-	-	-	0	0	-
No	-	-	-	-	424	96.8	-	-	-	-	381	96.5	-	-	-	-	43	100	-
**Retinopathy:**	-	-	-	-	-	-	-	-	-	-	-	-	-	-	-	-	-	-	0.38
Yes	-	-	-	-	7	1.6	-	-	-	-	7	1.8	-	-	-	-	0	0	-
No	-	-	-	-	432	98.4	-	-	-	-	389	98.2	-	-	-	-	43	100	-
**Therapy:**	-	-	-	-	-	-	-	-	-	-	-	-	-	-	-	-	-	-	0.01*
Insulin therapy	-	-	-	-	249	56.7	-	-	-	-	233	58.8	-	-	-	-	16	37.2	-
Medication therapy	-	-	-	-	190	43.3	-	-	-	-	163	41.2	-	-	-	-	27	62.8	-
**Previous amputation:**	-	-	-	-	-	-	-	-	-	-	-	-	-	-	-	-	-	-	0.36
Yes	-	-	-	-	89	20.2	-	-	-	-	78	19.7	-	-	-	-	11	25.6	-
No	-	-	-	-	350	79.8	-	-	-	-	318	80.3	-	-	-	-	32	74.4	-
**Hypertension:**	-	-	-	-	-	-	-	-	-	-	-	-	-	-	-	-	-	-	0.11
Yes	-	-	-	-	151	34.0	-	-	-	-	141	35.6		-	-	-	10	23.3	-
No	-	-	-	-	288	66	-	-	-	-	255	64.4		-	-	-	33	76.7	-
**Cardiovascular disease:**	-	-	-	-	-	-	-	-	-	-	-	-	-	-	-	-	-	-	0.08
Yes	-	-	-	-	27	6.2	-	-	-	-	27	6.8	-	-	-	-	0	0	-
No	-	-	-	-	412	93.9	-	-	-	-	369	93.2	-	-	-	-	43	100	-
**Dyslipidaemia:**	-	-	-	-	-	-	-	-	-	-	-	-	-	-	-	-	-	-	0.13
Yes	-	-	-	-	20	4.4	-	-	-	-	20	5.1	-	-	-	-	0	0	-
No	-	-	-	-	419	95.6	-	-	-	-	376	95	-	-	-	-	43	100	-
**Tuberculosis:**	-	-	-	-	-	-	-	-	-	-	-	-	-	-	-	-	-	-	0.42
Yes	-	-	-	-	6	1.4	-	-	-	-	6	1.5	-	-	-	-	0	0	-
No	-	-	-	-	433	98.6	-	-	-	-	390	98.5	-	-	-	-	43	100	-
**HIV:**	-	-	-	-	-	-	-	-	-	-	-	-	-	-	-	-	-	-	0.85
Positive	-	-	-	-	18	4.1	-	-	-	-	16	4	-	-	-	-	2	4.7	-
Negative	-	-	-	-	421	95.9	-	-	-	-	380	96	-	-	-	-	41	95.4	-
**Cancer:**	-	-	-	-	-	-	-	-	-	-	-	-	-	-	-	-	-	-	0.06
Yes	-	-	-	-	2	0.5	-	-	-	-	1	0.3	-	-	-	-	1	2.3	-
No	-	-	-	-	437	99.5	-	-	-	-	395	99.8	-	-	-	-	42	97.7	-
**Other comorbidities:**	-	-	-	-	-	-	-	-	-	-	-	-	-	-	-	-	-	-	0.07
Yes	-	-	-	-	29	6.4	-	-	-	-	29	7.3	-	-	-	-	0	0	-
No	-	-	-	-	410	93.6	-	-	-	-	367	92.7	-	-	-	-	43	100	-
**Other surgeries:**	-	-	-	-	-	-	-	-	-	-	-	-	-	-	-	-	-	-	0.82
Yes	-	-	-	-	45	10.3	-	-	-	-	41	10.4	-	-	-	-	-	9.3	-
No	-	-	-	-	394	89.7	-	-	-	-	89.6	-	-	-	-	-	-	90.7	-
**Intra-hospital referral:**	-	-	-	-	-	-	-	-	-	-	-	-	-	-	-	-	-	-	< 0.001*
Yes	-	-	-	-	426	97.5	-	-	-	-	389	98.5	-	-	-	-	38	88.4	-
No	-	-	-	-	11	2.5	-	-	-	-	6	1.5	-	-	-	-	5	11.6	-
**Ulcer classification:**	-	-	-	-	-	-	-	-	-	-	-	-	-	-	-	-	-	-	< 0.001*
Yes	-	-	-	-	35	8	-	-	-	-	6	1.5	-	-	-	-	29	67.4	-
No	-	-	-	-	404	92	-	-	-	-	390	98.5	-	-	-	-	14	32.6	-
**Level of previous amputation:**	-	-	-	-	-	-	-	-	-	-	-	-	-	-	-	-	-	-	0.20
Minor	-	-	-	-	30	6.8	-	-	-	-	28	7.1	-	-	-	-	2	4.7	-
Major	-	-	-	-	55	12.5	-	-	-	-	46	11.6	-	-	-	-	9	20.9	-
No previous amputation (%)	-	-	-	-	354	80.7	-	-	-	-	322	81.3	-	-	-	-	32	74.4	-
**Current amputation:**	-	-	-	-	-	-	-	-	-	-	-	-	-	-	-	-	-	-	0.54
Minor	-	-	-	-	96	21.9	-	-	-	-	85	21.5	-	-	-	-	11	25.6	-
Major	-	-	-	-	343	78.1	-	-	-	-	311	78.5	-	-	-	-	32	74.4	-
**Final disposition:**	-	-	-	-	-	-	-	-	-	-	-	-	-	-	-	-	-	-	< 0.001 *
Home	-	-	-	-	245	65	-	-	-	-	238	71	-	-	-	-	7	16.7	-
Referring hospital	-	-	-	-	132	35	-	-	-	-	97	29	-	-	-	-	35	83.3	-
**Admission diagnosis:**	-	-	-	-	-	-	-	-	-	-	-	-	-	-	-	-	-	-	< 0.001 *
Gangrene	-	-	-	-	143	32.6	-	-	-	-	123	31.1	-	-	-	-	20	46.5	-
Infection	-	-	-	-	20	4.6	-	-	-	-	20	5.1	-	-	-	-	0	0	-
Sepsis	-	-	-	-	68	15.5	-	-	-	-	54	13.6	-	-	-	-	14	32.6	-
CLTI	-	-	-	-	62	14.1	-	-	-	-	58	14.7	-	-	-	-	4	9.3	-
Non-healing ulcer	-	-	-	-	132	30.1	-	-	-	-	127	32.1	-	-	-	-	5	11.6	-
Necrotising fasciitis	-	-	-	-	14	3.2	-	-	-	-	14	3.5	-	-	-	-	0	0	-

HbA1c, haemoglobin A1c; HIV, human immunodeficiency virus; s.d., standard deviation; NMAH, Nelson Mandela Academic Hospital.

* , The distribution of the characteristic was significantly different between the two hospitals.

Approximately 56.7% of the participants were on insulin and oral medication and 43.3% on oral medication therapy. In Livingstone Hospital, 58.8% were on insulin therapy and oral medication, while in NMAH sample, only 37.2% were on insulin therapy and oral medication (*p* = 0.01).

The diagnoses at admission were gangrene (32.6%), infections (4.6%), sepsis (15.5%), critical limb threatening ischaemia (CLTI) (14.1%), non-healing ulcers (32.1%) and necrotising fasciitis (3.2%). In Livingstone Hospital, gangrene (31.1%) and non-healing ulcers (31.8%) were the most common diagnoses on admission, and in the NMAH, the most common diagnoses on admission were gangrene (46.5%) and sepsis (32.6%, *p* ≤ 0.001).

Patients from both Livingstone Hospital and NMAH had been referred to other departments within each hospital such as dietetics, physiotherapy, orthotics and prosthetics, occupational therapy and optometry for additional health services. The final disposition differed between Livingstone Hospital and NMAH. In Livingstone Hospital, 71% of the patients were discharged to go home, while in the NMAH sample, 15.7% were discharged to go home and the rest (83.3%) were sent back to the referring hospitals (*p* < 0.001).

### Survival experiences of diabetic-related lower extremity amputation between Livingstone Hospital and Nelson Mandela Academic rural hospital

**FIGURE 1:**
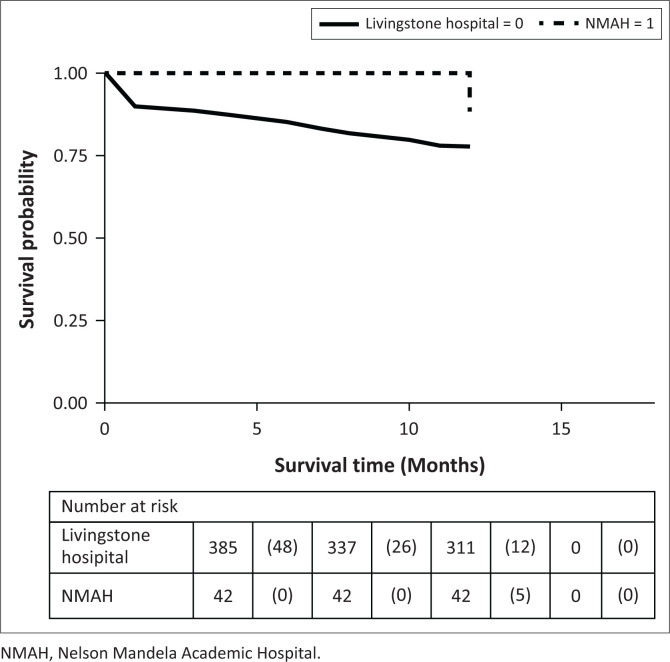
Kaplan–Meier survival estimates of patients at Livingstone Hospital and Nelson Mandela Academic rural hospital in year 1.

Overall, 40% (173/429) patients died by year 3. At 1 year, 22% (86/386) of patients in the urban Livingstone Hospital had died compared to 12% (5/43) of patients at rural NMAH. We found no difference in the 1-year survival experiences between diabetic-related lower extremity amputees in the urban Livingstone Hospital and the rural NMAH (*p* = 0.10; Figure 1).

**FIGURE 2:**
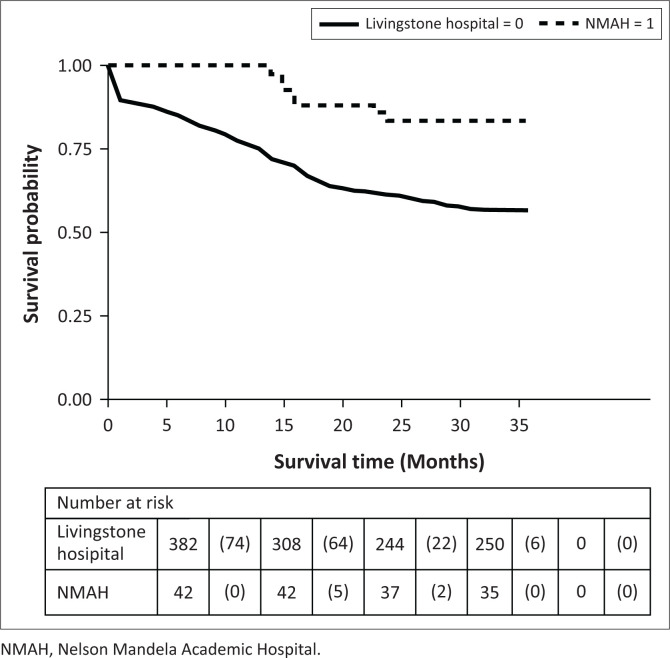
Kaplan–Meier survival estimates of patients at Livingstone Hospital and Nelson Mandela Academic rural hospital in year 3.

At year 3, 43% (166/386) of the patients in the urban Livingstone Hospital had died compared to 16% (7/43) patients at rural NMAH. We found a significant difference in the 3-year survival distributions of the two groups (*p* = 0.001; Figure 2).

### Factors associated with 3-year survival among patients with diabetic-related lower extremity amputations

Three-year survival was significantly associated with geographical location, with an HR of 0.38 (95% CI: 0.18–0.83), which showed reduced mortality for NMAH residents. A percent increase in HbA1C increased the risk of mortality by 4%, HR 1.04 (95% CI: 1.00–1.09), and major amputation almost doubled the risk of mortality, HR 1.83 (95% CI: 1.18–2.87). The absence of nephropathy HR 0.50 (95% CI: 0.25–1.00), non-cardiovascular disease HR 0.45 (95% CI: 0.28–0.74), no human immunodeficiency virus (HIV) HR 0.49 (0.43 [95% CI: 0.26–0.90]) and absence of other comorbid HR 0.42 (95% CI: 0.26–0.67) reduced the risk mortality. Patients at rural NMAH hospital had a lower risk of death, with an HR of 0.38 (95% CI: 0.18–0.83; Table 3).

**TABLE 3:** Factors associated with 3-year survival experiences, Cox regression model.

Characteristic	Univariate analysis			Multivariate analysis		
	HR	*p*	95% CI	HR	*p*	95% CI
**Geographical location**						
Urban	1.00	-	-	1.00	-	-
Rural	0.31	0.00*	0.15–0.67	0.38	0.02*	0.18–0.83
**HbA1C (%)**	1.06	**0.01**	1.01–1.10	1.04	0.03*	1.00–1.09
**Neuropathy**						
Yes	1.00	-	-	1.00	-	-
No	0.52	< 0.001*	0.37–0.72	0.93	0.75	0.61–1.42
**Nephropathy**						
Yes	1.00	-	-	1.00	-	-
No	0.34	< 0.001*	0.18–0.62	0.50	0.05*	0.25–1.00
**Therapy**						
Insulin and oral	1.00	-	-	1.00	-	-
Oral medication only	0.79	0.13	0.58–1.07	0.81	0.20	0.59–1.12
**Previous amputation**						
Yes	1.00	-	-	1.00	-	-
No	0.63	0.01*	0.45–0.89	0.77	0.19	0.53–1.13
**Cardiovascular disease**						
Yes	1.00	-	-	1.00	-	-
No	0.28	< 0.001*	0.18–0.43	0.45	< 0.001*	0.28–0.74
**Dyslipidaemia**						
Yes	1.00	-	-	1.00	-	-
No	0.49	0.02*	0.27–0.87	0.69	0.23	0.37–1.27
**HIV**						
Positive	1.00	-	-	1.00	-	-
Negative	0.50	0.02*	0.28–0.89	0.49	0.02*	0.27–0.90
**Other comorbidities**						
Yes	1.00	-	-	1.00	-	-
No	0.32	< 0.001*	0.20–0.50	0.42	< 0.001*	0.26–0.67
**Amputation**						
Minor amputation	1.00	-	-	1.00	-	-
Major amputation	2.00	0.06*	0.98–4.09	1.84	0.01	1.18–2.87

HbA1c, haemoglobin A1c; HR, human resource; HIV, human immunodeficiency virus; CI, confidence interval.

* , The characteristic was significantly associated with mortality.

## Discussion

We found high 3-year mortality rate among diabetic patients with LEAs. Similar mortality rates were reported in other studies (Beeson et al. 2023; Gök et al. 2016), which found high 5-year mortality rates following LEA (Beeson et al. 2023; Gök et al. 2016). Several studies compared diabetic-related complications between urban and rural diabetic populations (Akinlotan et al. 2021; O’Connor & Wellenius 2012; Tai et al. 2020). Some studies compared the survival of diabetic patients and non-diabetic patients (Eliasson et al. 2011; Tayek 2012). Our findings are contradictory to the results from studies in Australia and in the United States, which indicated that people living in rural areas had double the incidence of DM and were highly likely to have poor health outcomes and mortality (Andrus et al. 2004; *Rural, regional and remote health A study on mortality (2nd edition)* Phillips 2007). However, we also found that patients in the rural hospital had longer hospital stay compared to patients in the urban hospital. People living in rural areas often experience health disadvantages because of geographical barriers such as poor road infrastructure, lack of access to specialist care in local hospitals, poor self-management and lack of health education programmes (Somasundram et al. 2019). The conflict in the study findings may be attributed to differences in geographical location and selection bias because of the limited number of patients from the rural hospital.

Not surprisingly, high blood glucose and major amputation were significantly associated with higher risk of mortality. Patients free of comorbidities had a lower risk of mortality. We found a clinically significant reduction in mortality rate of 23% although not statistically significant among patients with no previous amputations than those with a previous amputation. Uncontrolled HbA1c have been associated with poor wound healing and poor surgical outcomes, high incidence of re-amputation and death (Nayak & Kirketerp-Møller 2016).

Patients without nephropathy had reduced risk of mortality compared to those with a nephropathy. Microvascular complications of DM are long-term high blood glucose conditions that affect the small blood vessels, resulting in neuropathy, retinopathy and nephropathy (Lavery et al. 2010). Previous studies have shown that the prevalence of nephropathy is high in diabetic patients residing in rural areas (Chireshe, Manyangadze & Naidoo 2024). The risk of mortality was lower among HIV-negative patients. Even with improvements of antiretroviral treatments in people living with HIV, the presence of diabetes and HIV greatly increases the risk of cardiovascular disease and mortality (Chireshe et al. 2024). This suggests that healthcare providers should closely monitor people living with HIV and diabetes and other comorbidities that increase mortality.

Major amputation increased patient’s risk of mortality. Perioperative mortality is largely influenced by the level of the amputation. A higher mortality rate has been shown for above knee amputation compared to below knee amputation. Poor survival rates have been linked to factors such as ageing, multiple comorbidities, above knee amputations, post-operative amputation and post-amputation mobility and ambulation (Kristensen et al. 2012). Diabetic patients with major amputation are a high-risk population that require close monitoring and specialised care.

### Study limitations

The comparison of survival distribution between the rural NMAH and urban Livingstone Hospital should be interpreted with caution because of a small number of patient’s medical files that were available in the rural hospital. We were cautious on drawing a definite conclusion on this comparison. Rural hospitals have poor infrastructure and lack specialised care services and we therefore expected high mortality rates in the rural hospital. The small number of patient’s medical files in the rural hospital may have contributed to the unexpected study findings. Incomplete data on comorbidities in the medical files and an unknown number of medical files that were at a storage space away from the hospital site we could not access limited the number of patients included in the study at the rural NMAH hospital, and this may have introduced selection bias. This study used data collected retrospectively, bias limiting the number of variables to test association with mortality. Study findings may not be generalised to other settings. More studies comparing survival experiences between rural and urban patients with diabetic-related LEAs are needed.

## Conclusion

The study compared survival rates and factors associated with 3-year mortality in patients with diabetic-related LEAs between Livingstone urban hospital and NMAH. Establishing multidisciplinary care teams for patients with diabetic-related LEAs and other comorbidities and aggressive monitoring and management of blood glucose and comorbidities may reduce the risk of mortality.
